# Effective engagement of community health workers in primary eye care in India

**Published:** 2018-07-31

**Authors:** Prem Kumar SG, Shubhrakanti Bhattacharya, Pankaj Vishwakarma, Sabitra Kundu, Elizabeth Kurian

**Affiliations:** 1 Manager: Research, Mission for Vision, Mumbai, India.; 2 Senior Manager: Programme Development, Mission for Vision, Mumbai, India.; 3 Head: Programme Impact, Mission for Vision, Mumbai, India.; 4 Head: Programme Development, Mission for Vision, Mumbai, India.; 5 Chief Executive Officer: Mission for Vision, Mumbai, India.

**Active and sustained involvement of existing community health workers in primary eye care service can help South Asian countries tackle a major challenge in the region - lack of trained human resources.**

**Figure:**
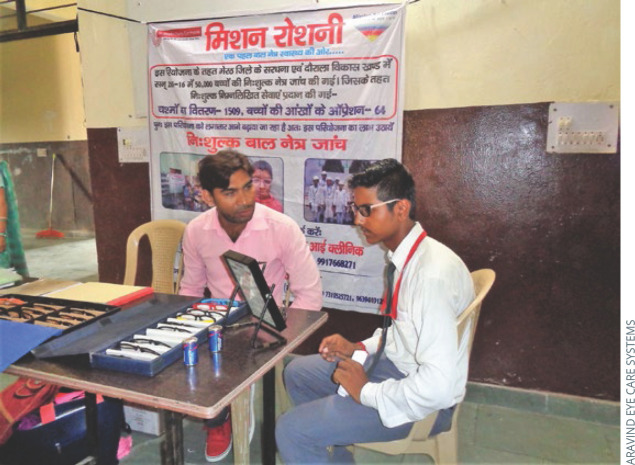
School eye health project in Meerut district, Uttar Pradesh. INDIA

Bourne and colleagues from the Vision Loss Expert Group estimated that there are close to 253 million visually impaired individuals worldwide in 2015 of which 14.2% are blind. India contributed about one-fifth to the global magnitude of blindness.^1^,^2^ Despite the recent gains, cataract and refractive errors continue to constitute about 75% of moderate to severe vision impairment (MSVI) globally.^1^

The WHO's Universal Eye Health: A Global Action Plan 2014 – 2019 has prioritised the development and maintenance of a sustainable workforce for the provision of comprehensive eye care services as a key action for reaching the objective of universal eye health.^3^ Lack of trained human resources is recognised as one of the greatest challenges to reducing the prevalence of avoidable blindness in India.^4^ Given the inadequacy of human resources in healthcare settings including eye care, Mishra and colleagues have strongly argued for a greater role and engagement of community level volunteers like Accredited Social Health Activist (ASHA) provided they are appropriately trained and sensitised.^5^

## Mission for Vision's (MFV) experience in engaging community health workers in primary eye care

### *Anganwadi* workers (AWW)

*Anganwadi* centres are government-run mother and child care centres in the villages of India. The *anganwadi* workers are women selected from local communities who ensure antenatal and postnatal care for pregnant women, nursing mothers and immediate diagnosis and care for new born children. They are also agents of social change and mobilise community support for better care of young children, girls and women.^6^

MFV's engagement with AWWs began in 2015 with a joint initiative with Dr Shroff's Charity Eye Hospital (SCEH), New Delhi. It involved provision of eye health services to children, enrolled in schools and those out-of-schools in Sardhana and Daurala, in Meerut district of Uttar Pradesh. As part of this two-year initiative called Mission Roshni, a total of 89,433 children aged 0 to 16 years were screened for eye conditions. Children in schools and madrasas (an institution for the study of Islamic theology and religious law) were screened by optometrists whilst those out of school and aged 0 to 6, by trained AWWs. Approvals from officials at the local Integrated Child Development Services (ICDS) office were obtained for the training and involvement of AWWs in the community eye health (CEH) project.

The training programme for AWWs was tailor-made to suit the project objectives and were standalone exercises. AWWs were paid a monetary incentive of INR. 250/- per each surgical referral and INR. 2/- for each child mobilised for eye screening. A total of 662 teachers and 302 AWWs were trained. Of the total child screenings done, 16,544 (18.5%) were by AWWs. A total of 3,161 (3.5%) were identified with refractive errors and 3,147 (99.6%) received corrective glasses. Ten children were identified with low vision and 139 (0.2%) were identified for surgical treatment.^7^

Active engagement of AWWs helped in generating awareness and counselling of parents to seek appropriate treatment for their children. Working with AWW was a challenge particularly given their varied backgrounds and competencies which impacted the overall performance.

### Accredited Social Health Activist

ASHAs are community health workers instituted by the government of India's Ministry of Health and Family Welfare as a part of the National Rural Health Mission.^8^ ASHAs serve as a bridge between the healthcare system and rural populations. They motivate women to give birth in hospitals, bring children to immunisation clinics, encourage family planning, and treat basic illness and injury with first aid.^9^

In two of India's north-eastern states, Mizoram and Meghalaya, community eye health (CEH) initiatives were undertaken with the help of ASHAs. First, in collaboration with Synod Hospital, Aizawl, trained ASHAs conducted door-to-door eye screenings in all the 118 villages of Aizawl and Kolasib districts. Adults aged 50 years or older, who were suspected or self-reported to have eye health issues were advised to visit a local eye camp organised in their respective villages. At these camps, optometrists screened patients for eye conditions including cataracts. Those diagnosed with cataracts and having a visual acuity (VA) of <6/24 were referred to the base hospital for further medical assessment. A total of 158 trained ASHAs helped in organising 143 eye screening camps. 5,445 individuals were screened of which 935 eyes were operated.

**Figure:**
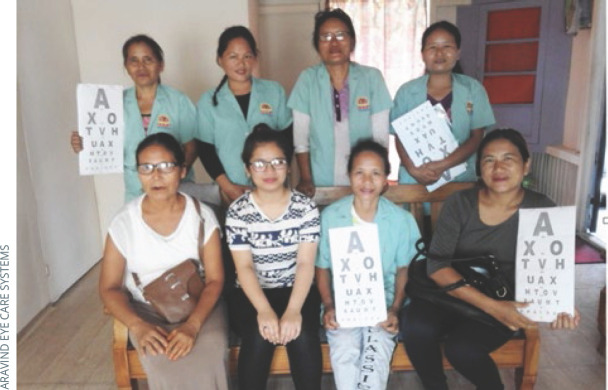
ASHA training for community eye health project in Kolasib district, Mizoram. INDIA

The Meghalaya Integrated Eye Health Project was initiated in collaboration with Society for Promotion of Eye Care and Sight (SPECS), Shillong in 2017 in two districts: East and West Jaintia Hills. The target of this project was to screen 9,000 individuals living in small clusters of villages across the two districts. A total of 135 ASHAs were trained and door-to-door campaigns were conducted to educate and screen the local population for eye conditions. All suspected or self-reported cases were referred to local eye outreach camps, where optometrists screened for potential eye conditions. ASHAs also accompanied the patients from their residence to the camp-site. A total of 122 outreach camps were organised with active participation of ASHAs. 13,790 adults were screened and referred by ASHAs, of whom 1,038 (7.5%) were diagnosed with cataracts and 405 eyes were operated upon.

In both the states, approvals from officials at the District Medical & Health Office (DMHO) were sought for the training and involvement of ASHAs. All trained ASHAs were paid a one-time monetary incentive of INR. 1,000/- for screening and referral to eye health camps.

Involving ASHAs in the CEH projects had helped in creating awareness in the community and improved demand for eye health services. However varying levels of motivation and willingness of ASHAs was a challenge as some felt this was an additional burden on them. Transport is a major issue in Meghalaya which made it difficult for both ASHAs and patients to travel to eye care centres.

### *Mahila Arogya Samiti* (MAS) workers

MAS workers are community-based women's groups who serve local communities in health planning and action under the National Urban Health Mission framework.^10^ Vision centres (VCs) set up by Mission For Vision in association with Sightsavers and Kolkata Municipal Corporation in Kolkata city are the first point of interface for this urban population to address their eye health needs. There are nine VCs in the urban slums of Kolkata to cater to the eye health needs of underprivileged populations.

In 2016, local MAS were co-opted to improve demand for eye services in the region and over the last couple of years 504 MAS workers were trained. These training programmes were tailor- made to suit the needs of the project. No monetary incentives were provided to MAS for their role in the project. In the last two years, the nine VCs catered to about 40,000 patients, of which MAS accounted for about a quarter of all referrals. Actively engaging MAS workers has contributed to an increase in the uptake of primary eye health services, and ensured provision of appropriate follow-up services to the patients. High rates of attrition was the main challenge while working with MAS workers.

## Conclusion

Blindness and visual impairment continues to be a major public health problem in India. Availability and easy access to primary eye care services is therefore essential for elimination of avoidable blindness. The advantage of integrating eye health within community health and development initiatives with the engagement of local community workers promotes increase in uptake of primary eye care services. Active and sustained involvement of existing community health workers in primary eye care service provision is a win-win solution, specifically in geographies which are difficult and remote.
